# Targeting the PI3K signaling pathway in *KRAS* mutant colon cancer

**DOI:** 10.1002/cam4.591

**Published:** 2015-12-29

**Authors:** Suntaek Hong, SoYoung Kim, Hye Youn Kim, Myunghee Kang, Ho Hee Jang, Won‐Suk Lee

**Affiliations:** ^1^ Lee Gil Ya Cancer and Diabetes InstituteGachon UniversityIncheonKorea; ^2^ Department of SurgeryGil Medical CenterGachon UniversityIncheonKorea; ^3^ Gachon Medical Research InstituteGil Medical CenterIncheonKorea; ^4^ Department of PathologyGil Medical CenterGachon UniversityIncheonKorea

**Keywords:** BKM120, colon cancer, combination therapy, KRAS, PIK3CA

## Abstract

Metastatic colorectal cancer (CRC) patients with v‐Ki‐ras2 Kirsten rat sarcoma viral oncogene homolog (KRAS) mutations are resistant to monoclonal antibody that targets the epidermal growth factor receptor such as cetuximab. BKM120 targets phosphatidylinositide‐3‐kinase (PIK3CA), but it is unknown whether BKM120 can reverse cetuximab resistance in KRAS mutant CRC. Human CRC cell lines with KRAS mutations (DLD‐1, HCT116, and LoVo) were used to test the effect of cetuximab, BKM120, and cetuximab plus BKM120 on cell proliferation in vitro and in vivo. BKM120 reduced cell proliferation in a concentration‐dependent manner in the LoVo (PI3KCA wild type) as well as the HCT116 and DLD1 cells (that carry a PI3KCA mutation). BKM120 only inhibited ERK phosphorylation in LoVo cells (PIK3CA wild type), but not in DLD1 or HCT116 cells at a concentration of 1 *μ*mol/L. Treatment with cetuximab and BKM120 significantly reduced the growth of xenograft tumors originating from KRAS mutant cells compared with cetuximab alone (*P* = 0.034). BKM120 may overcome cetuximab resistance in colon cancer cells with KRAS mutation.

## Introduction

Despite recent therapeutic advances, the prognosis of patients with *KRAS* mutant metastatic colorectal cancer (CRC) remains dismal with an average median overall survival (OS) of approximately 13.5 months 1. Activation of the phosphatidylinositide‐3‐kinase (PI3K)/serine/threonine‐specific protein kinase (AKT)/mammalian target of rapamycin (mTOR) pathway has been implicated in the growth and progression of various cancers, as well as resistance to standard anticancer therapies 2. PI3Ks are lipid kinases that promote various cellular processes, including proliferation and survival 3. PI3K pathway activation is caused by the presence of a gain of function mutation in the *PI3KCA* gene, and recently BKM120, a drug that targets the PI3K pathway, was used to treat stage I non‐small cell lung cancer patients with tumors showing PI3K pathway activation.

The benefit of adding cetuximab to first‐line or second‐line irinotecan‐containing chemotherapy regimens has been addressed in two prospective trials—cetuximab combined with irinotecan in first‐line therapy for metastatic CRC (CRYSTAL) 4 and oxaliplatin plus cetuximab in first‐line treatment of metastatic CRC (OPUS)^5^. In these studies, patients with KRAS mutations had no survival benefit with the addition of cetuximab to FOLFIRI or FOLFOX treatment in both trials [4, 5].

Preclinical and clinical data suggest that mutations in the *KRAS* gene may influence the response to PI3K/AKT/mTOR inhibitors and may mediate resistance to these agents 6. Therefore, in this study, we characterized the *PI3KCA* and *KRAS* mutational status of several human colon cancer cell lines. We then examined the therapeutic effects of BKM120 in combination with cetuximab in these cell lines both in vitro and in a xenograft model of this malignancy.

## Materials and Methods

### Ethics statement

All experiments involving animals were approved in advance by the Animal Ethics Committee at Lee Gil Ya Cancer and Diabetes Institute, Gachon University, Incheon, Korea and were carried out in accordance with the Australian code of practice for the Care and Use of Animals for Scientific Purposes.

### Cells and reagents

The human CRC‐derived cell lines DLD1, HCT116 and LoVo were purchased from the American Type Culture Collection and were maintained in RPMI‐1640 culture medium (WelGENE, Daegu, Korea) supplemented with 10% fetal bovine serum (FBS; WelGENE) and 1% penicillin‐streptomycin solution (WelGENE) at 37°C in a humidified atmosphere with 5% CO_2_. Cetuximab (C225; Erbitux; purchased from Merck, Darmstadt, Germany) was used at a final concentration of 5 mg/mL. BKM120 (200 mg) was purchased from Chemie Tek (Indianapolis, IN).

### Cell proliferation assay

Cell proliferation was evaluated using CCK‐8 assay kit (cat. # CK04‐01; Dojindo, Kumamoto, Japan) according to the manufacturer's specifications. Colon cancer cells were cultured at a density of 1 × 10^4^ cells/mL in 96‐well plates. The cells were incubated for 24 h, and then treated with BKM120 and cetuximab diluted in culture media at the indicated concentrations for 48 h at 37°C. After addition of CCK‐8 solution for 4 h, the absorbance was measured at 450 nm using a microplate reader.

### Cell based enzyme‐linked immunosorbent assay for adherent cells

We used immunoassay kits for human phospho‐AKT (S473) and human phospho‐ERK1 (T202/Y204)/extracellular‐signal‐regulated kinases (ERK2) (T185/Y187) (cat. # KCB887, KC1018; R&D, Minneapolis, MN). Cultured colon cancer cell lines (HCT116, DLD1 and LoVo) were seeded in 96‐well plates at 30,000 cells/well. When appropriate, the cells were serum starved for 4 h and treated with BKM120 and cetuximab for 1 h. After treatment, the cells were fixed by replacing the medium with 100 *μ*L of 4% formaldehyde in phosphate‐buffered saline (PBS) for 20 mins at room temperature and were washed 3 times with PBS. Each wash step was performed for 5 mins with gentle shaking. The cells were then exposed to quenching buffer with 0.6% H_2_O_2_ for 20 mins, followed by blocking buffer with 10% FBS for 1 h. Subsequently, the cells were incubated overnight with the primary antibodies (total AKT/ERK and phospho‐AKT/ERK antibody) at 4°C. The following day cells were washed 3 times with PBS for 5 mins, incubated with secondary antibody (horseradish peroxidase [HRP]‐conjugated antibody and alkaline phosphatase [AP]‐conjugated antibody, dilution 1:100) in PBS for 1 h at room temperature, and washed 3 times with PBS for 5 mins. Subsequently, the cells were incubated with 50 *μ*L of a solution containing a fluorogenic substrate for HRP and AP for 1 h at room temperature in the dark. The reaction was measured with two channels (A_540/600_ and A_360/450_) using the fluorescence plate reader Victor^3^ (1420‐011; Perkin Elmer, Boston, MA). The readings at 600 nm represent the amount of phosphorylated AKT/ERK in the cells, while readings at 450 nm represent the amount of total AKT/ERK in the cells.

### Evaluation of apoptosis by Caspase‐3 colorimetric assay

For analysis of caspase‐3 activity, we used caspase‐3/CPP32 Assay kit (K105‐100) according to the manufacturer's instructions (BioVision, San Francisco, CA, USA) in homogenized cell lysates using ELISA. The emission intensity of the sample was expressed as absorbance 405 nm.

### In vivo xenograft study

Male BALB/c nude mice, 4–6 weeks old, were obtained from Orient Bio, Inc (Seongnam, Korea). Mice were implanted subcutaneously with 1.0 × 10^7^ LoVo cells in Matrigel (Becton Dickinson Franklin Lakes, NJ). Tumors were allowed to grow to a size of 300–500 mm^3^, and animals were then randomly distributed to 4 groups for treatment and control. Cetuximab was administered at 10 mg/kg intraperitoneally twice a week. BKM120 was administered at 50 mg/kg intraperitoneally 3 times a week for 25 days. The longitudinal (*L*) and transverse (*W*) tumor dimensions were measured twice a week and used to calculate the tumor volume (*V*) with the following formula: *V* = (*L* × *W* ^2^)/2. After killing the mice, tumor tissues were collected, fixed with 10% formalin, and embedded in paraffin wax. Mice were housed under pathogen‐free conditions and maintained on a 12 h light/12 h dark cycle, with food and water supplied ad libitum. The animal protocol was reviewed and approved by the Institutional Animal Care and Use Committee at Gachon University.

## Results

### Combined treatment of BKM120 and cetuximab reduces cell viability in vitro

**Figure 1:**
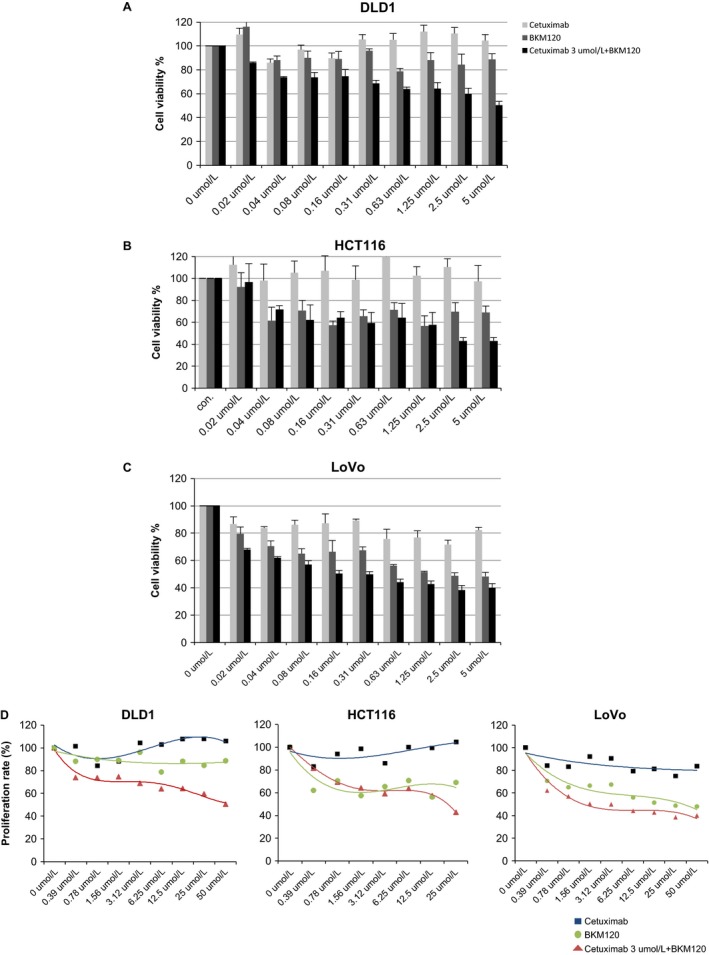
Effects of combination therapy on the proliferation of human colon cancer cells. Colon cancer cells (1 × 10^4^ cells/well) were cultured in a 96‐well plate and treated with halving serial dilutions of 50 *μ*g/mL cetuximab, 10 *μ*g/mL BKM120, or combined treatments of halving serial dilutions of 10 *μ*g/mL BKM120 with 3* μ*g/mL cetuximab for 48 h. Cells were treated with 10 *μ*L CCK‐8 solution/well and incubated for 4 h at 37°C. The amount of formazan dye was measured by absorbance at 450 nm with a microplate reader. (A) DLD1; (B) HCT116; and (C) LoVo cells. Error bars indicate SD;* n* = 3 technical replicates of a representative experiment.

To understand the importance of PI3K signaling in KRAS mutated colon cancer, we selected human colon cell lines based on mutation status of KRAS and PI3K genes (Table 1) Cell proliferation was measured using the CCK‐8 assay after a 48 h incubation with BKM120, cetuximab, or a combination of both. BKM120 reduced cell proliferation in a concentration‐dependent manner in the LoVo (*PI3KCA* wild type) as well as the HCT116 and DLD1 cells (that carry a *PI3KCA* mutation). DLD1 cells (carrying the *PI3KCA* E545K mutation) were less sensitive to the effects of BKM120, while HCT116 (carrying the *PI3KCA* H1047R mutation) and LoVo cells required a concentration of at least 12.5 to reach 50% decrease in cell proliferation (Fig. 1).

**Table 1:**
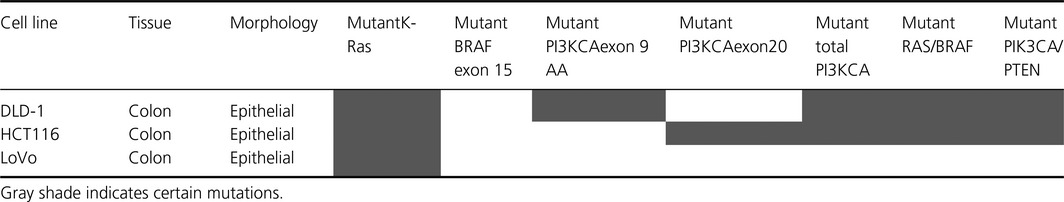
Characteristics of used colon cancer cell lines in this study

### Inhibition of AKT and ERK signaling pathways by BKM120 and cetuximab in colon cancer cells

**Figure 2:**
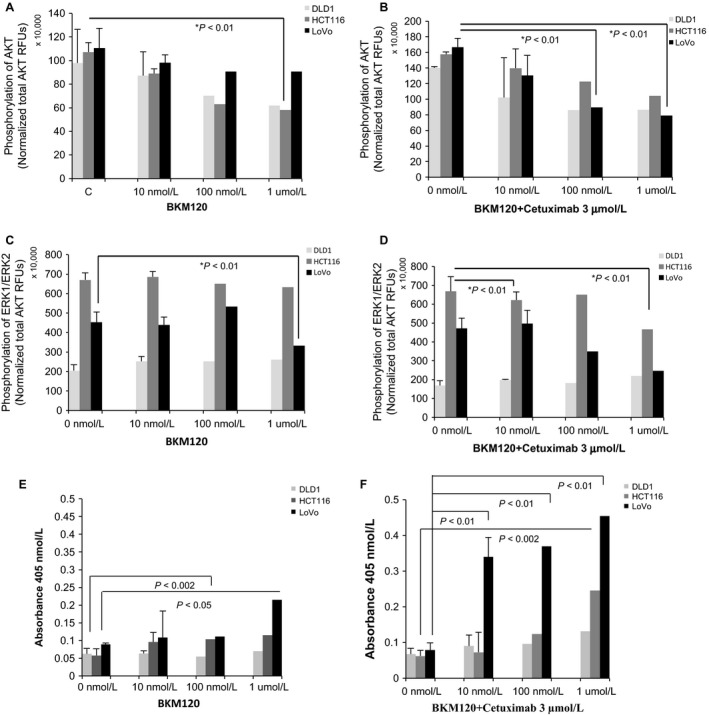
Inhibitory activity of BKM120 for AKT/ERK phosphorylation. Results obtained with different dilutions of BKM120 and cetuximab are shown. Phosphorylation of AKT after cells were treated with a halving serial dilution of 10 *μ*g/mL BKM120 (A) and a combination treatment consisting of halving serial dilutions of 10 *μ*g/mL BKM120 with 3* μ*g/mL cetuximab (B) was determined. Phosphorylation of ERK with a halving serial dilution of 10 *μ*g/mL BKM120 (C) and combination treatment consisting of halving serial dilutions of 10 *μ*g/mL BKM120 with 3* μ*g/mL cetuximab (D) was also determined. Error bars indicate SD;* n* = 3 technical replicates of a representative experiment. (E) and (F) Activation of caspase‐3 was checked with colorimetric assay after treatment of BKM120 alone or combination with cetuximab as indicated concentrations.

AKT is activated by phosphorylation of serine^473^, and ERK is activated by phosphorylation of threonine^185^/tyrosine^187^ and threonine^202^/tyrosine^204^. Because these signals are critical for proliferation of cancer cells, we assessed whether phosphorylation of ERK or AKT were changed after treatment with BKM120, cetuximab, or both for 1 h. BKM120 inhibited AKT phosphorylation in all of the colon cancer cell lines tested at a concentration of 1 *μ*mol/L (Fig. 2A). Combination treatments inhibited AKT phosphorylation more effectively than either drug alone in all of the colon cancer cells (Fig. 2B). However, BKM120 only inhibited ERK phosphorylation in LoVo cells (*PI3KCA* wild type), but not in DLD1 or HCT116 cells at a concentration of 1 *μ*mol/L (Fig. 2C). Combination treatments inhibited ERK phosphorylation more effectively in HCT116 and LoVo cells at the same concentration (Fig. 2D).

### Activation of Caspase‐3 after treatment with BKM120 and cetuximab

To address the inhibitory mechanism of BKM120 and cetuximab on tumor growth, we examined the activation of caspase‐3 using colorimetric assay. BKM120 alone activated caspase‐3 with very low level in colon cancer cells (Fig. 2E). However, combined treatment of BKM120 and cetuximab markedly increased the activity of caspase‐3 in cells (Fig. 2F). This result indicates that BKM120 and cetuximab cooperate to inhibit the tumor growth by inducing apoptosis.

### BKM120 cooperates with cetuximab to inhibit tumor growth in vivo

**Figure 3:**
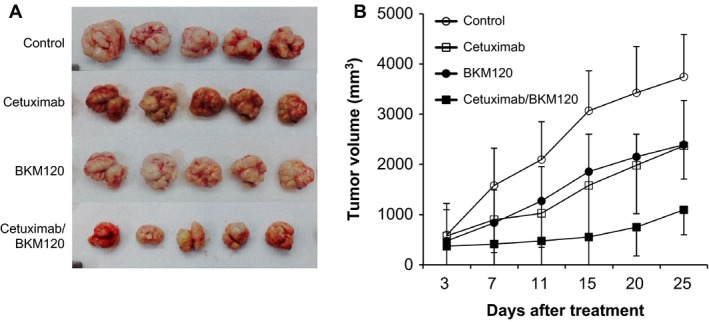
Effect of combination therapy on the in vivo growth of colon cancer cells. (A) Representative images of LoVo xenograft tumors removed surgically on day 25 demonstrating the difference in tumor volumes between control and combination drug treatment groups. (B) BALB/c nude mice were injected subcutaneously with LoVo colorectal cancer cells. After the tumor size reached 300–500 mm^3^, mice were assigned into 1 of 4 groups that were treated with phosphate‐buffered saline (PBS), cetuximab alone, BKM120 alone or a combination of cetuximab and BKM120. The mice were treated twice per week with an intraperitoneal injection of 10 mg/kg cetuximab and/or 3 times a week with an intraperitoneal injection once daily of 50 mg/kg BKM120. Tumor dimensions were measured twice a week with a digital caliper and these were used to calculate the tumor volume. Error bars indicate SD;* n* = 5 mice per group.

To validate the inhibitory activity of BKM120 on colon cancer, we generated in vivo xenograft model using nude mice. We assessed whether BKM120 treatment could retard tumor growth in vivo. Combined cetuximab and BKM120 treatment significantly reduced the growth of LoVo tumors compared to cetuximab alone (Fig. 3A and B). Four weeks after transplantation, the mean tumor volume in the control group was 4500 mm^3^, while it was only 1000 mm^3^ in the BKM120 combination treatment group (Fig. 3B). With respect to the rate of tumor growth, the mean control tumor volume increased from 3000 mm^3^ at week 1 to 4500 mm^3^ at week 4, while tumors treated with cetuximab or BKM120 alone increased from 2000 mm^3^ to 3000 mm^3^ over the same period (Fig. 3B). However, there was a significantly greater reduction in tumor growth in mice treated with combined BKM120 and cetuximab. The cetuximab and BKM120 combination in PI3KCA wild‐type LoVo cells demonstrated a greater inhibitory effect on cell growth compared with cetuximab or BKM120 alone treatment for 25 days (LoVo, 10 mg/kg cetuximab vs. 10 mg/kg cetuximab + 50 mg/kg BKM120, mean growth = 62.14% vs. 39.67%, difference = 22.47%; cetuximab; *P* = 0.034) (Fig. 3B).

## Discussion

The optimization of colon cancer treatment is complicated by the concomitant development of tumor escape mechanisms. Recent improvements in the treatment of colon cancer, such as antiepidermal growth factor receptor (EGFR) therapy using cetuximab, are limited by resistance arising from oncogenic mutations in signaling receptors and pathways [7.] In CRC, 40% of patients carry a *KRAS* mutation, 15% carry a *BRAF* mutation, and 20% carry a *PI3KCA* mutation [7.] These patients do not respond to available anti‐EGFR therapies and it is considered to be a disease subset equivalent to the triple negative form of breast cancer.

In this study, we demonstrated that the combination of BKM120 and cetuximab significantly enhanced antitumor activity against *KRAS* mutant/*PI3KCA* wild‐type LoVo cells. BKM120 binds to the ATP‐binding site of the lipid kinase in both a competitive and noncompetitive manner. Preclinical studies on glioma and myeloma revealed that BKM120 had different activity in different cell lines [8, 9, 10], and cell lines harboring *PI3KCA* oncogenic mutations had a greater sensitivity to BKM120 compared to those with the wild‐type *PI3KCA* gene 11. A recently published study showed that the presence of a concomitant *KRAS*‐activating mutation reduced sensitivity to BKM120, reflecting previous clinical findings with mTOR inhibitors such as everolimus 12. However, in this study, we found that the growth of *KRAS* mutant xenograft tumors was significantly inhibited by combined BKM120 and cetuximab treatment, while this combination was not effective in *PI3KCA* mutant cell lines. To the best of our knowledge, this is the first report to show that BKM120 might overcome cetuximab resistance in *KRAS* mutant/*PI3KCA* wild‐type CRCs.

Combinatorial anticancer therapy with Mitogen‐activated protein kinases (MAPK) inhibitors was administered in cases of resistance to the mTORC1 inhibitor rapamycin. mTORC1 inhibition leads to the activation of the ERK/MAPK cascade. However, PI3K inhibition using LY294002 reduced rapamycin‐induced ERK activation 13. Furthermore, NVP‐BKM120 inhibited mTOR downstream activation, but induced the phosphorylation of AKT and ERK in *KRAS* mutant gastric cancer cells 14. Our data demonstrate that PI3K inhibition with BKM120 leads to p‐AKT downregulation, but not ERK phosphorylation, and instead actually reduces levels of p‐ERK in LoVo cells. Additionally, treatment of BKM120‐activated caspase‐3 in colon cancer cell lines. This caspase‐3 activation was followed by induction of apoptosis, as has been observed in other human tumor cells with these agents 15. Decrease in PI3K/AKT and MAPK/ERK pathways caused FOXO transcription to induce the cell cycle arrest and apoptosis in pancreatic cancer 16.

The efficacy of BKM120 has also been assessed in phase I and II clinical trials. Of note, among 35 triple‐negative breast cancer patients, one experienced a confirmed partial response and 7 had stable disease for at least 8 months 8. Although the results from such studies support the hypothesis that BKM120 may help to decrease the burden of cancer, the overall results from preclinical studies still remain obscure [8, 6]. Although phase III study of BKM120 in human epidermal growth factor receptor 2 (HER2)‐negative metastatic breast cancer is ongoing, there are currently only phase I trials for BKM120 in advanced CRC 17. These studies demonstrated the safety of class I PI3K inhibition in patients with advanced cancers. However, it is too early to determine the activity in the treatment of metastatic CRC. Further phase II and III trials are warranted.

Recent studies have reported that inhibition of the PI3K/AKT pathway increases the efficacy of chemotherapeutic agents in human malignancies including colon cancers [18, 19]. Gynecologic malignancies with *PI3KCA* mutations treated with PI3K/AKT/mTOR inhibitors demonstrated a higher response rate than *PI3KCA* wild‐type malignancies 20. A subgroup of patients with ovarian cancer with simultaneous *PI3KCA* and *MAPK* mutations responded to PI3K/AKT/mTOR inhibitors, suggesting that not all patients demonstrate resistance when the MAPK pathway is concomitantly activated 20. The results of this study indicate that the addition of BKM120 may overcome cetuximab resistance in *KRAS* mutant CRC by targeting the PI3KCA pathway in patients with *PI3KCA* wild‐type cancers.

## Conclusions

We demonstrated that BKM120 can increase the efficacy of cetuximab in *PI3KCA* wild‐type/*KRAS* mutant CRC by inducing apoptosis.

## Conflict of Interest

None declared.
